# Nanosensitive optical coherence tomography to monitor corneal burn and treatment response in vivo

**DOI:** 10.1364/BOE.599649

**Published:** 2026-06-12

**Authors:** Eanna Johnston, Sergey Alexandrov, Rajib Dey, Ellen Donohoe, Aoife Canning, Thomas Ritter, Martin Leahy

**Affiliations:** 1 Tissue Optics and Microcirculation Imaging (TOMI) Facility, School of Natural Sciences, University of Galway, Galway, Ireland; 2 Regenerative Medicine Institute (REMEDI), Discipline of Advanced Therapeutics, School of Medicine, University of Galway, Galway, Ireland; 3 CURAM, SFI Research Centre for Medical Devices, University of Galway, Galway, Ireland

## Abstract

Chemical burns account for 10–22% of ocular injuries, with alkali burns being the most frequent and severe. There is a significant need for treatment of these injuries, as well as an effective imaging method to monitor both the injury and treatment progression. Currently, there are limited options for monitoring the burn and treatment response of the sub-micron structures within the corneal tissue *in vivo* and non-destructively, due to factors such as the resolution limit of optical imaging systems. Optical coherence tomography (OCT) is a non-invasive imaging technique that provides high-resolution, cross-sectional imaging of biological tissue at good imaging depths. Due to the diffraction limit, OCT resolution is limited to the micron scale, preventing monitoring of nanoscale changes. Nanosensitive optical coherence tomography (nsOCT) overcomes this limitation by recovering high spatial frequency information through spectral encoding, enabling assessment of sub-micron tissue features. Here, we present variations of nsOCT with improved sensitivity for detecting nanoscale tissue changes. These methods were applied to a corneal alkali burn model and a treatment model using mesenchymal stromal cell-derived extracellular vesicles. Correlation of axial spatial frequency profiles (ASFP) and mapping of ASFP differences enabled sensitive detection of nanoscale structural changes post-injury and during treatment progression. A statistically significant disruption of the sub-micron structure was observed after injury, followed by partial restoration during the reparative phase. The proposed methods provide improved sensitivity to nanoscale tissue changes and may support monitoring of cell therapy and other medical applications for diagnostic purposes.

## 1. Introduction

Globally, there are on average sixty million incident cases of ocular injuries per year [1]. Approximately 10–22% of these injuries are due to alkali burns, with two-thirds of reported cases originating from workplace accidents [2]. Unlike acid burns, when the cornea is damaged, the lipophilic alkali ions denature proteins found in the stroma and can cause further damage towards the anterior chamber of the eye. Because of this, alkali burns can penetrate as far as the iris and the crystalline lens of the eye, causing widespread inflammation [3,4]. Investigating the corneal stroma is therefore an important factor when looking at alkali burns within the eye. The collagen matrix of the stroma is a key part of corneal transparency, and any disruption to this structure can decrease transparency and increase the light scattering within the cornea, reducing light that could otherwise reach the retina [4]. The scale of this issue means that there is a strong requirement for an effective treatment method, alongside a robust imaging method to monitor both damage progression, and treatment effectiveness. Recently, work has been conducted to investigate the effectiveness of mesenchymal stromal cell-derived extracellular vesicles (MSC-EVs) as a method to reduce corneal inflammation initiated by alkali injury by the upregulation of anti-inflammatory immune cells, namely regulatory T cell generation [5]. It has been previously found that the role of EVs has a significant impact on the contribution of the anti-inflammatory effects of MSCs [6,7]. The final effectiveness of this treatment can be seen through monitoring regulatory T cell production and an increase in reparative anti-inflammatory macrophages. However, this is only obtained at the post-mortem stage with no information about nanostructural changes during treatment progression being measured [5]. Direct visual inspection of the anterior segment in a slit lamp examination is the only routinely conducted clinical examination for ocular injuries to investigate stromal opacification, oedema, and epithelial loss in the cornea [8]. These examinations are quick, affordable, and provide good macroscopic information, but can be highly observer-dependent, give limited depth information, and give no quantitative information about nanoscale changes that occur due to ocular injury [8]. It is therefore important that a non-invasive, non-destructive imaging method is developed to perform *in vivo* imaging for effective injury and treatment assessment of the subcellular structure.

Several optical imaging methods have been used to investigate the structure of the cornea, such as optical elastography [9], brillouin microscopy [10], and multiphoton imaging [11]. These imaging methods are limited by either their lateral resolution being unable to detect sub-micron structures, or their imaging depth being insufficient for any realistic *in vivo* imaging. A solution to this is to use OCT due to its high resolution and imaging depth capabilities. Introduced in 1991 [12], OCT rapidly became the standard of care for retinal imaging in ophthalmology [13,14]. Since then, it has been applied to various other areas of medicine such as cardiology [15–17], dermatology [18–20], and dental imaging [21,22]. OCT is based on low-coherence interferometry, where the optical path length difference between the reference beam, and the sample beam lies within the coherence length of the light source. Originally, in time domain OCT (TD-OCT), this was achieved through mechanical movement of the reference arm to vary the path length difference [12]. For spectral domain OCT (SD-OCT), this is typically achieved by using a near-infrared broadband light source, with the wavelength contributions of the recombined beam being detected with a spectrometer, creating an interferogram. Fourier-domain OCT (FD-OCT) techniques were introduced by Fercher et al. [23] and took over from TD-OCT once both swept-source OCT (SS-OCT) and spectral-domain OCT (SD-OCT) were show to have an improvement in signal-to-noise ratio (SNR) roughly equivalent to the number of discreet wavelengths over which the noise was divided [24–26]. This interferogram is Fourier transformed, creating a depth profile in the axial direction (A-line). Through raster scanning of the sample, these A-lines can be produced in a defined field of view, creating a three-dimension volume made up of two-dimensional cross-sections (B-frames) [27]. Unlike most imaging methods, the axial and lateral resolution of these volumes is decoupled. The axial resolution is dependent on the bandwidth of the source, and the lateral resolution is inversely proportional to the numerical aperture of the objective lens. FD-OCT techniques allow for both good imaging depth, typically in the range of 3–5 mm, and good axial resolution, typically in the range of 6–15 μ m in air, with images being collected in real-time and at a high SNR. While these values are good enough to detect macroscopic changes occurring in tissue, the depth profiles generated contain no information about nanoscale changes that occur due to the diffraction limit of the system.

Several methods have been developed to improve the resolution and sensitivity of OCT. Efforts have been made to increase the lateral and axial resolution of OCT through the use of super-continuum lasers and high-precision optics. These systems allow for high axial and lateral resolutions in the range of 1-2 μ m [28–30]. While this is a significant increase in resolution compared to commercially available systems, it is still not high enough to detect any nanoscale changes in tissue structure due to the diffraction limit of the system. An emerging technique in OCT system development is the use of high numerical aperture objective lenses to achieve lateral resolutions down to 1.0 μ m. Modalities such as optical coherence microscopy (OCM) [31,32], full-field OCT (FF-OCT) [33–35], and line-field OCT (LF-OCT) [36–38] use this set-up to generate en face images to the cellular level to image the fine structure of tissue. Micro-optical coherence tomography ( μ OCT) is a high-resolution, non-invasive, imaging modality offering micron-level, real-time, 3D visualisations of tissue microstructures, with significantly higher resolution (often 1–2 μ m) compared to standard OCT [39–42]. Mazlin et al. developed an FF-OCT system that allowed for a high lateral resolution of 1.7 μ m. This system could detect several structures in the cornea that conventional OCT is unable to detect, such as the Bowman’s layer, sub-basal plexus, and structures within the epithelial layer [43]. However, the imaging depth of FF-OCT is limited, and its resolution limit still prevents it from gaining information about sub-micron structures in the tissue. Another paper by Khan et al. developed a blue-light OCM system with a lateral resolution of 1.6 μ m. This system could detect epithelial and endothelial cells within the cornea, as well as structures within the corneal stroma [44]. Similarly, this system sacrifices imaging depth for resolution and cannot detect nanoscale changes in the structure. Several new imaging modalities have emerged to improve the sensitivity of OCT imaging to the nanoscale. An emerging technique known as inverse spectroscopic OCT (IS-OCT) can detect nanoscale changes in the range of approximately 30–40 nm by calculating a mass density correlation parameter in tissue [45]. Techniques that investigate the changes in speckle content over time in images such as dynamic OCT have also been found to detect sub-micron structural features within cells [46]. The phase variation within OCT spectral interferograms can also be extracted in techniques such as phase-sensitive OCT, where structural changes of less than 1 nm can be detected [47].

Nanosensitive optical coherence tomography (nsOCT) is a recently developed technique by Alexandrov et al. where unresolved high spatial frequency information relating to nanoscale structures is reclaimed during OCT processing [48,49]. This is based on spectral encoding of spatial frequencies, which allows for monitoring nanoscale structural changes and super-resolution imaging [50–54]. Each wavelength component of the FD-OCT signal relates to a specific axial spatial frequency with a range of frequencies being defined by the bandwidth of the illumination source. Each scattering point can be defined as having a range of spatial frequency energy contributions, with the dominant spatial period being mapped to its corresponding voxel. By doing this, nanoscale information, which is lost in conventional OCT processing, can now be visualised, giving a sensitivity to structural alterations at the nanoscale. This method has found success in detecting nanoscale functional and structural changes in both *ex vivo* and *in vivo* tissue and allows for conventional OCT systems to be able to map cellular and subcellular nanoscale changes in tissue [55–60]. Spatial frequency correlation mapping OCT (sf-cmOCT) has also been described recently [61]. This method calculates the Pearson correlation coefficient between axial spatial frequency information generated at two different points. This method was developed to see if nsOCT sensitivity could be improved through correlating depth resolved axial spatial information generated at two time points. It was found that the small changes in biological tissue occurring during occlusion of human skin tissue could be detected through mapping correlation coefficients.

Previously, nsOCT was found to be an effective imaging modality to detect structural changes in corneal tissue associated with corneal burn and treatment responses [57,62]. This paper is primarily an extension of the study by Lal et al., where only the dominant spatial period of the cornea following wound healing was investigated. In this paper, we apply variations of nsOCT in an attempt to increase the imaging sensitivity by looking at all captured spatial periods and introduce an MSC-EV treatment. These methods include the previously mentioned sf-cmOCT, and a new method, which calculates the difference between spatial frequency contributions at two regions of interest. These methods are verified using reference Bragg grating samples and applied to a corneal alkali burn model.

## 2. Methodology

### 2.1. Corneal burn model

All animal procedures were performed under the approval of the Animal Care Research Ethics Committee of the University of Galway, and conducted under individual authorisation licenses from the Health Products Regulatory Authority (HPRA) of Ireland (Project Authorisation Number: AE19125/P113). All animals were housed and cared for under standard operating procedures of the Animal Facility at the Biomedical Sciences Biological Resources Unit, University of Galway. All mice were purchased from Charles River Laboratories. Corneal burns were applied to mice under anaesthesia with isoflurane. A round piece of filter paper soaked in 1.0 M sodium hydroxide was applied to the central cornea of the left eye for thirty seconds, and washed with a PBS solution. OCT images of the cornea and unaffected contralateral eye were then collected. A detailed outline of the burn and treatment model can be found in Donohoe et al. [5].

### 2.2. Experimental setup

Two different treatment groups were investigated in this study. One group applies the MSC-EV treatment subconjunctivally (N = 7), while the other acts as a PBS control (N = 5), also injected subconjunctivally. OCT images were taken on days 1, 3, and 7 after initial corneal burn, with a control group of undamaged corneas taken as the control. For all OCT imaging, a commercial TELESTO III spectral domain OCT system from Thorlabs, Inc. was used. This system contains a 1300 nm centred broadband light source, with a spectral range from 1180 to 1415 nm. This allows for an axial resolution of 5.5 μ m, and an imaging depth of 3.6 mm in air. The objective lens used has a numerical aperture of 0.055 allowing for a lateral resolution of 13 μ m. Images were acquired at a rate of 76 kHz, with a FOV of 1×1 mm in the lateral direction and 500 pixels taken in each lateral direction. For each 3D volume acquired, 30 en face images through the depth of the cornea were taken during analysis.

### 2.3. Corneal thickness measurements

An edge detection algorithm based on dynamic programming was implemented to detect the anterior and posterior surfaces of the cornea within each OCT volume. This uses a graph-based shortest-path method similar to those used for OCT layer segmentation [63]. More detail on the edge detection method used can be found in the Supplement 1.

### 2.4. Nanosensitive OCT

This section outlines the theory behind nsOCT; however, the inverse scattering theory based on the first Born approximation can be found in Alexandrov et al. [49]. Nanosensitive OCT (nsOCT) reclaims high spatial frequency information that is typically lost during conventional OCT processing. This is achieved through spectral encoding of spatial frequencies, which examines the wavelength distribution at each voxel of the image in the Fourier domain. The relation between axial spatial frequencies and wavelength can be seen in Eq. (1).

(1) $$\nu_{z}=\frac{n(\cos\theta +\cos\alpha )}{\lambda }$$

Where $\nu_{z}$ is the axial spatial frequency, θ and α are the illumination and scattering angles respectively, n is the refractive index of the sample medium, and λ is the light source wavelength [50]. In OCT, these two angles can be approximated to zero, simplifying the equation to a one-to-one relation between wavelength and axial spatial frequency, as seen in Eq. (2) [50].

(2) $$\nu_{z}=\frac{2n}{\lambda }$$

The range of spatial frequencies obtained by the detector can then be described by Eq. (3).

(3) $$\Delta \nu =\frac{2n\Delta \lambda }{\lambda_{1}\lambda_{2}}$$

where Δλ is the source bandwidth and $\lambda_{1}$ , $\lambda_{2}$ are the lower and upper limits of the source respectively. This relates the complex amplitude axial spatial frequency signals and wavelengths found within the spectral interference signal generated by FD-OCT. These axial spatial frequencies can be rewritten in terms of axial spatial periods, which relate to structural size, by Eq. (4).

(4) $$H_{z}=\frac{1}{\nu_{z}}$$

When looking at the SD-OCT system used in this study, the spatial frequencies range from 4.70×10^−3^ to 5.64×10^−3^ ${\text{mm}}^{-1}$ , giving a spatial period range of 591.7 nm to 709.2 nm when n=1.00 . If we take the physical refractive index to be 1.376 for the cornea, then the range of captured axial spatial periods ranges from 430.0 nm to 515.4 nm. After OCT processing of the signal including k-space linearisation and DC signal removal, the wavelength dependent interferogram is rescaled in terms of spatial frequencies or periods based on the relations in Eq. (4). To determine the energy contributions of available spatial periods, the interference spectrum is then divided into N windows. For example, a tapered cosine window such as a Tukey window. Each of these windows is zero padded and will represent a specific spatial period as defined by the spatial period range and its position on the interference signal axis. For this study, and the system used, 15 zones were taken when dividing the interferogram into Tukey windows. The number of zones taken can be adjusted depending on the application and system used as there is balancing between spatial and spectral resolution required. The frequency bandwidth of each zone can be described as

(5) $$\delta \nu_{z}=\frac{\Delta \nu_{z}}{N}$$

where N is the number of windows. Each window within the spectrum is then Fourier transformed to give a depth profile (A-line). The contribution of the spatial frequency energy into each point within the depth profile is calculated from all N depth profiles. What is formed at each depth of the A-line is known as an axial spatial frequency profile (ASFP) or period profile (ASPP) which gives comprehensive information about spatial frequency or period energy contributions [50]. It is these spatial period contributions that relate to the nanoscale structures found in tissue. There is the fundamental tradeoff between depth resolution and spectral resolution, which has been discussed in our previous papers [48,52]. In this paper we form and analyse the en face nsOCT images where we do not have degradation in spatial (lateral) resolution. The nsOCT image can then be formed as a colour map of some informative parameter such as the dominant or mean spatial period. Alongside this, a 4 x 4 median spatial filtering kernal and a threshold value of 2.0 was taken for noise suppression. These values can be adjusted depending on the application.

### 2.5. Spatial frequency correlation OCT

After the ASFP has been formed, profiles can be compared depending on the application. The same profile can be monitored at two time moments, one profile can be compared with all other profiles within the object, or two profiles of two different objects can be compared. One way to compare these profiles is to calculate the Pearson correlation coefficient between two profiles. This method is based on the correlation calculations used is correlation mapping OCT (cmOCT) [64], but rather than comparing the intensity signal found in conventional OCT, the ASFPs are compared, demonstrating the nanoscale structural changes that occur in biological tissue. By mapping the Pearson correlation coefficient at each voxel, the linear relationship between two points can be described. Areas of low correlation indicate that changes are occurring in the axial spatial frequency or period contribution, with a high correlation indicating little to no change. The Pearson correlation coefficient can be described by the equation

(6) $$r=\frac{\Sigma (x_{i}-\bar{x})(y_{i}-\bar{y})}{\sqrt{\Sigma {\left(x_{i}-\bar{x}\right)}^{2}\Sigma {\left(y_{i}-\bar{y}\right)}^{2}}}$$

where x and y are the two ASFP being correlated, with $\bar{x}$ and $\bar{y}$ being their means. For this study, a profile at one time point is taken and correlated with all other profiles at the same time point. This has advantages over the dominant spatial period method of nsOCT, as not only does it contain information about how the nanoscale structure changes over time, but also provides insight into how the order of the structure varies throughout the cornea. The generated profile underwent the same processing steps as all other profiles found within each OCT volume for consistency, and was selected near the central depth of the cornea. It was found that the location of the reference profile did not impact the generated results significantly. However areas where high noise is present were avoided during profile selection.

### 2.6. Difference in axial spatial frequency profiles

**Fig. 1.**
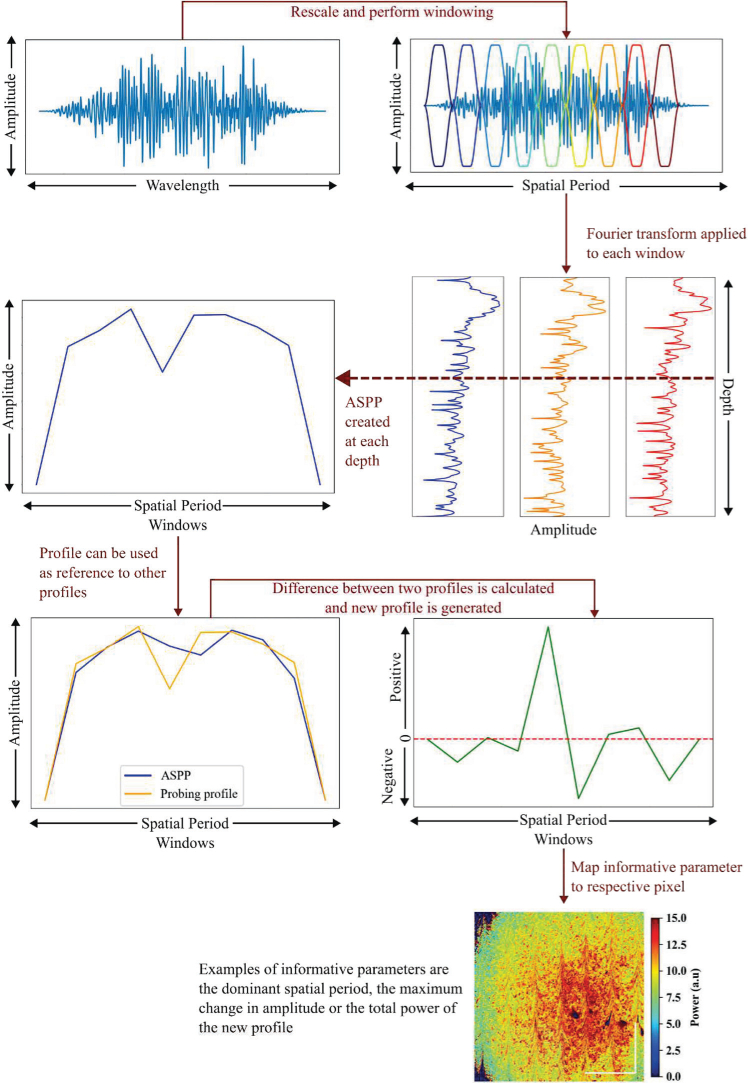
Flow chart of difference in ASFP algorithm processing.

A new method is presented in this paper, where the difference between two ASFPs is calculated, forming a new profile that contains comprehensive information about the nanoscale structural differences between two objects. Using this method, two ASFPs are taken, for example, a healthy cornea reference profile, and an injured cornea profile, and the difference between the two is found, providing a new ASFP. This can be described as

(7) $$\Delta I_{i}(\nu_{z})=I_{p}(\nu_{z})-I_{h}(\nu_{z})$$

where $I_{p}(\nu_{z})$ is a pathological profile, $I_{h}(\nu_{z})$ is the reference healthy profile, and $\Delta I(\nu_{z})$ is the new profile formed. Since axial spatial period contribution relates to structural sizes, the changes in sub-micron structural sizes can be monitored by visualising the contribution differences between two profiles. The new profile can then be colour mapped as some informative parameter. One visualisation method is to find the total absolute spatial frequency contribution difference as an integral of the new ASFP through the equation

(8) $$\Delta I_{\mathrm{int}}=\int_{\nu_{z,\min}}^{\nu_{z,\max}}\left|\Delta I_{i}(\nu_{z})\right|\;d\nu_{z}$$

where $\nu_{z,\min}$ and $\nu_{z,\max}$ are the lower and upper bounds of the axial spatial frequency bandwidth. It is expected that the pathological areas will be visualised with a higher $\Delta I_{\mathrm{int}}$ value, and the healthy areas will be visualised with a lower value. A flow chart of this process can be seen in Fig. 1. Other visualisation methods such as examining the positive and negative values separately, or mapping the new dominant or mean spatial period can also be mapped. Using this method, the variation of spatial frequency contribution was monitored by taking a reference healthy profile and subtracting it from profiles at all other time points. This will give comprehensive information about the nanoscale changes that occur during corneal injury and treatment progression. The power of each new ASFP was then calculated and colour mapped. The reference pixel selection differs for this method compared to sf-cmOCT. Here, a reference healthy profile is taken, and the difference is found between it and all other profiles at every time point. Rather than investigate the internal structural disorder of the cornea like in sf-cmOCT, we now want to look at how the axial spatial frequency contibutions differ from the healthy, uninjured cornea. This improves the diagnostic capabilities of nsOCT, as there is a direct comparison with the expected healthy structure. It also investigates the change in all captured axial spatial periods, as opposed to only the dominant structure. The selected reference profile also underwent the same processing steps as all other profiles.

## 3. Results & discussion

### 3.1. Validation of nsOCT

**Fig. 2.**
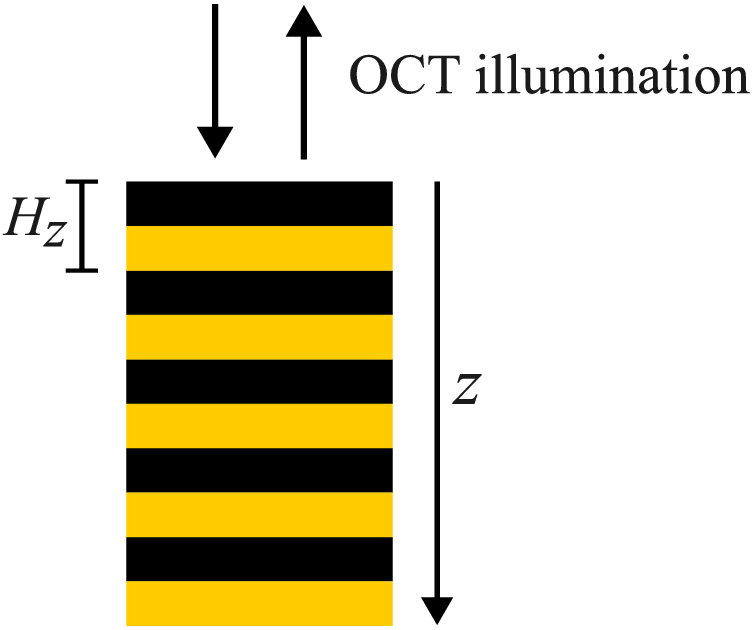
Schematic of Bragg gratings used for OCT imaging. $H_{z}$ represents the axial spatial period relating to the spacing between refractive index changes, with z being the total depth.

**Fig. 3.**
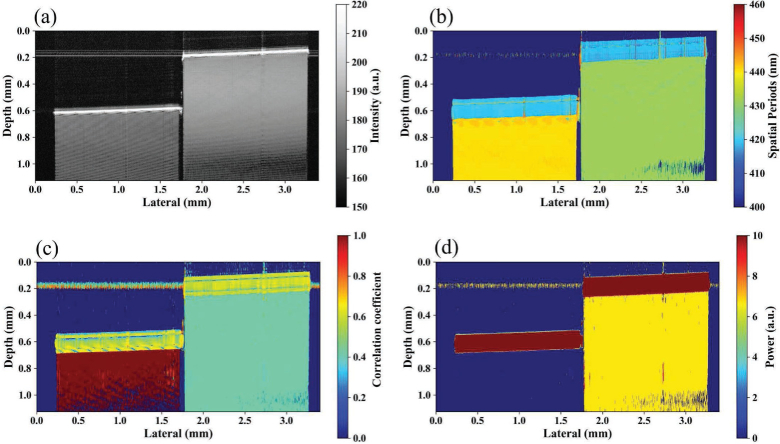
Experimental demonstration of (a) OCT, (b) nsOCT, (c) sf-cmOCT, and (d) difference in ASFP using Bragg gratings. For each image, the colour bar represents a different informative parameter. (a) represents an arbitrary intensity value of backscattered light, (b) represents the structural size in nanometers, (c) represents the Pearson correlation coefficient, and (d) represents the ASFP power change.

To validate the various nsOCT modalities presented, OCT images were taken of two Bragg gratings obtained from OptiGrate Corp. USA. These gratings contained internal periodic structures of 431.56 nm and 441.76 nm, respectively, with an average refractive index of 1.48. These contain well-defined periodic structures along depth caused by internal refractive index variations and help to demonstrate how nsOCT can be used to detect nanoscale changes in submicron structures. Figure 2 shows a simple schematic for these gratings. These gratings also contain an antireflective coating at the top surface, being represented by a different colour in each B-frame. In Fig. 3(a), it can be seen that conventional OCT cannot detect these structures due to high spatial frequency information containing information about nanoscale structures being lost during OCT processing. For each of the nsOCT modalities, different informative parameters are being mapped. When looking at the dominant spatial period generated through nsOCT processing within each structure in Fig. 3(b), the nanoscale difference between structures is visualised. For the correlation and difference in ASFP methods, a profile from the 441.76 nm grating was taken. In Fig. 3(c), the profile is correlated with all other profiles found within the image. It can be seen that there is a correlation coefficient of 1 for the grating where the reference profile was taken, and a lower coefficient for the profiles found in the other grating. For 3(d), the difference in axial spatial periods is visualised through calculating the total change in energy contribution of the new profile. We can see how the grating where the reference profile is taken goes to zero as there are no variations in the sub-micron structure, whereas the other grating visualises a change in the sub-micron structure.

### 3.2. Corneal macroscopic changes and thickness measurements

**Fig. 4.**
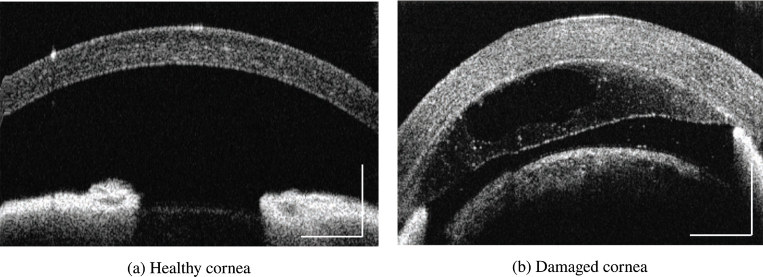
OCT B-frames of cornea before and after alkali burn. (a) Healthy cornea. (b) Damaged cornea. Scale bar – 250 μ m.

Next, the macroscopic structure of the cornea post-alkali injury and treatment was investigated using conventional OCT. Several features of note can be seen when comparing the healthy and damaged corneas in Fig. 4. There is a clear increase in corneal thickness that occurs, as well as the fusion of the iris to the posterior surface, and layers peeling from the posterior surface into the anterior chamber of the eye. In some cases, the damage reaches as far as the crystalline lens of the eye, due to the alkali denaturing proteins in the corneal stroma and causing damage to reach the anterior chamber. There is an increase in scattering within the cornea itself due to corneal oedema, causing opacification. By implementing the edge detection method described in the methodology, the corneal thickness of the healthy and burned cornea was found. Table 1 shows the corneal thickness increases between the injured corneas and the unaltered contralateral eye. This increase in corneal thickness is due to acute stromal oedema that occurs during alkali injuries.

**Table 1.** Comparison of healthy and injured corneal thickness with their change in mean thickness Δs in μ m.

Sample	Healthy	Injured	Δs
**S1**	150±21	349±28	200
**S2**	160±14	292±25	132
**S3**	151±7	295±39	144
**S4**	152±27	298±20	146
**S5**	141±21	393±34	252

### 3.3. sf-cmOCT and difference in ASFP results

**Fig. 5.**
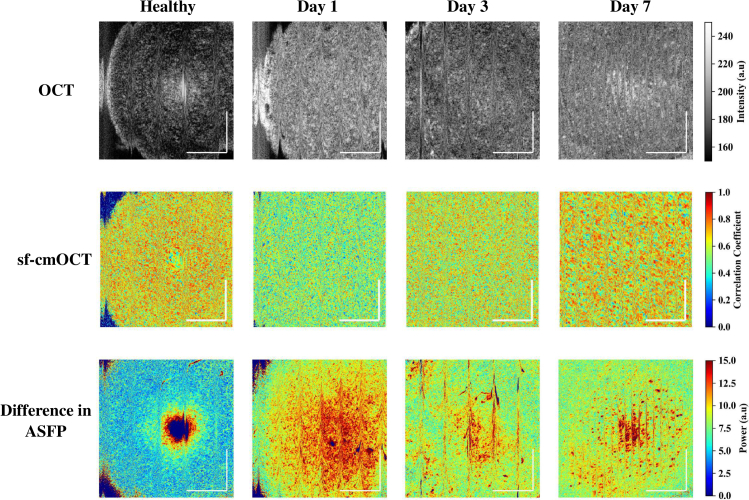
En face images of MSC-EV corneal treatment progression using conventional OCT, sf-cmOCT, and difference in ASFP method for healthy, day 1, day 3, and day 7. Scale bar – 300 µm.

**Fig. 6.**
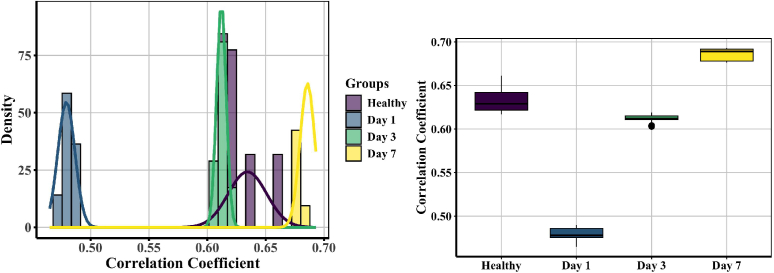
Pearson correlation coefficient distribution of MSC-EV treatment group.

**Fig. 7.**
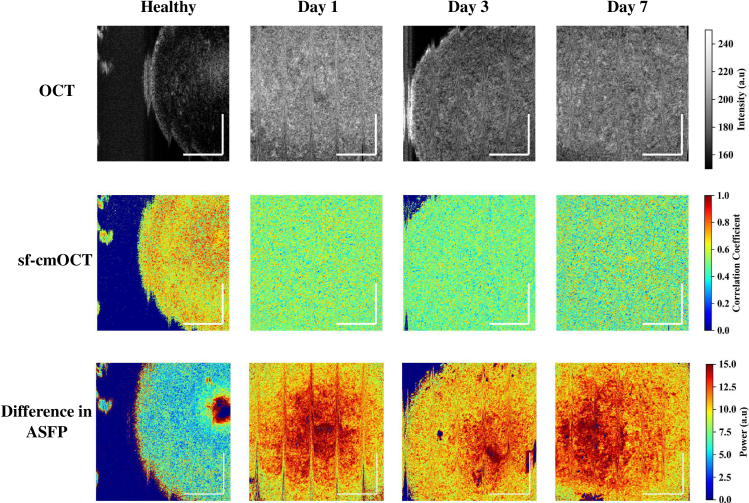
En face images of PBS control corneal progression using conventional OCT, sf-cmOCT, and difference in ASFP method for healthy, day 1, day 3, and day 7. Scale bar – 300 µm.

While conventional OCT can see these macroscopic structural changes, information about sub-micron changes in the tissue structure is lost during processing, giving no indication about how nanoscale structures are changing within the cornea over the course of treatment. To examine these changes, we compared the ASFP generated from nsOCT using both the spatial frequency correlation mapping and difference method mentioned in the methodology. For spatial frequency correlation, the reference ASFP was taken from one time point and correlated with all other profiles found at the same time point, mapping the Pearson correlation coefficient to the corresponding pixel. By doing this, we can observe the structural order within each cornea, with lower coefficients indicating structural disorder, and higher coefficients close to 1 indicating an ordered structure. Figure 5 shows images in the en face plane using this spatial frequency correlation mapping method. It can be observed that the correlation coefficient at day 1 drops significantly compared to the healthy cornea, and an increase in correlation coefficient can be seen at days 3 and 7 compared to day 1. A box plot and histogram for one of the seven samples was generated to better visualise this change in correlation coefficient over each day of treatment, as seen in Fig. 6. These plots were generated by taking the mean correlation coefficient at every depth of the cornea. An ANOVA test was performed to investigate the statistical significance of variance between each group, which showed that at a 95% confidence interval, the p-value was less than $10^{-10}$ between day 1 and day 7. The decrease in correlation coefficients in the injured cornea compared to the healthy cornea indicates that there is a weak linear relationship between the sub-micron structures found within the cornea. The increase in correlation coefficient as the treatment reaches day 7 indicates that the pro-reparative MSC-EV treatment is causing the stroma to regain the original structural order [5]. Enface images of the PBS control samples can be seen in Fig. 7, which show how the correlation coefficients remain low within the cornea by day 7.

**Fig. 8.**
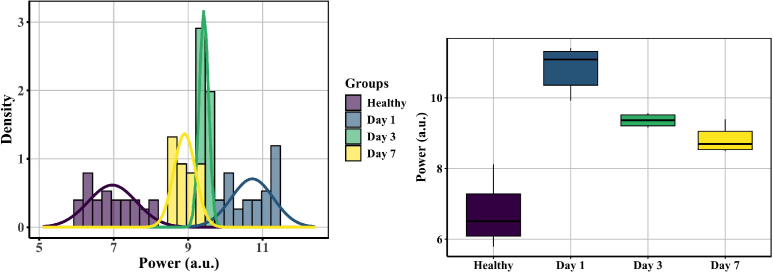
Spatial period distribution plots for MSC-EV treatment using difference in ASFP algorithm.

**Fig. 9.**
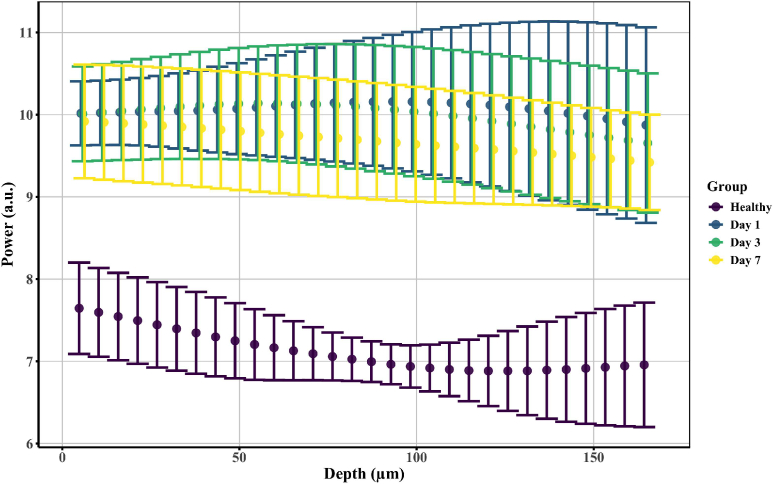
Mean ASFP power value and standard deviation of en face images through corneal depth of MSC-EV treated samples.

To monitor how the spatial frequency contributions between healthy, injured, and treated corneas change over time, the new algorithm based on calculating the difference between ASFPs was used. A healthy profile from the unaltered contralateral cornea was generated and used as a reference against all other profiles of all groups at every time point. A new profile was generated and then integrated to find the area under the curve, which contains the difference in spatial frequency contributions between the treated and healthy corneas. The en face images using this difference in ASFP method in Fig. 5 reveal qualitative information about the sub-micron structure, with better stability and qualitative information about pathological and treated regions throughout the cornea as the structures change in time. For day 1 treated corneas, the spatial frequency differences are high, indicating that the sub-micron structure of the damaged cornea has changed significantly. When monitoring the cornea at days 3 and 7, it can be seen that the difference in spatial frequency contributions decreases, revealing that the profiles at day 7 more closely reflect the healthy profiles compared to those found at day 1. This is visualised in Fig. 8, where the distribution of spatial frequency contribution differences can be seen, with statistically significant differences (p < $10^{-10}$ ) between all treatment time points. Here, the damaged cornea’s ASFP differences at day 1 increase significantly with a high standard deviation. The median value reduces as treatment progresses, and the structure becomes more ordered and stable as the standard deviation decreases. A plot of mean power vs depth was generated for all samples in each treatment group by taking the mean power value and standard deviation at each en face depth. When looking at the mean ASFP power values through the corneal depth in Fig. 9, there is a decrease in the mean between at day 1 compared to days 3 and 7, with the standard deviation in values at day 7 remaining lower compared to day 1. Occasionally, there is still an increase in the power value when day 1 is compared to day 7. However, these samples do show a decrease between day 7 and day 3, indicating that treatment may have only been effective by day 3 onwards. By day 7, this change has decreased due to the anti-inflammatory effects of the MSC-EV treatment [5]. En face images of the PBS control samples can be seen in Fig. 7, showing high spatial frequency differences by day 7. The Supplement 1 attached to this paper contains more graphical representations of the PBS control samples when calculating the ASFP power values and correlation coefficients. This supplement also contains tables of values for all samples imaged using both of the presented nsOCT methods.

### 3.4. Biological interpretation of nsOCT results and limitations

The change in the periodic structure between the healthy and injured corneas is likely linked to inflammation and swelling in the cornea caused by the activation of keratocytes within the stroma, which increases the size of nanoscale structures. It is known that the activation of keratocytes within the collagen matrix causes disruption between college fibrils, and the spacing between them becomes irregular. The inflammatory signalling cascade changes collagen fibril diameter, spacing due to stromal oedema, and arrangement, as well as proteoglycan and glycosaminoglycan networks [65–67]. Corneal transparency and wound healing are primarily governed by stromal ultrastructure, and not gross morphology. Pathological changes affecting optical transparency, mechanical integrity and wound healing trajectory originate at the nanoscale before macroscopic. Compared to standard OCT, nsOCT is highly sensitive to fibrillar organisation, ECM disruption and collagen spacing abnormalities, which occur before loss of visual acuity or stromal opacity. Non-invasive imaging that can capture these early changes may enable detection of sub-clinical injury prior to the manifestation of macroscopic signs. This can guide early therapeutic intervention, inform monitoring frequency, or stratify those likely to develop chronic fibrosis, preventing progression to loss of visual acuity. Other standard approaches such as slit-lamp microscopy specifically address the gross morphology and are insensitive to early/subtle stromal changes [65–67]. Ultrastructure assessment has traditionally required ex vivo microscopy. The nsOCT methods presented offer a non-invasive platform for repeated measurements for longitudinal corneal healing monitoring.

Due to the sensitivity to motion of OCT, some artifacts due to sample breathing are present during imaging. These can be seen in the en face images as vertical lines down the y-axis. However, it was found that environmental noise from operators and other equipment present had little to no effect during imaging. The centre of the healthy cornea en face images also contained high amounts of glare and saturation while imaging, with the corresponding A-lines masked while calculating the difference between ASFP as to not affect the quantitative analysis. These artifacts appeared to have little effect during sf-cmOCT processing. An assumption that is made is that the refractive index does not change throughout the cornea. It is known that the cornea contains multiple layers with changes in refractive index throughout of less than 0.03 [68–70]. This does not significantly affect the results generated using nsOCT due to the small change this would cause to the spatial period range. The refractive index also increases after alkali injury due to corneal hydration [71,72]. However, we assume the refractive index remains the same throughout injury and treatment for simplicity in calculations, as we cannot accurately measure the refractive index of the cornea post-injury. This paper is not to show the accurate spatial period measurements found within the cornea, only to detect these changes in nanoscale structures that occur during injury and treatment. Processing was performed using a quad-core Intel i7 2.8 GHz processor and 16 GB of memory. Processing of this data and plotting all 500 B-frames and 30 en face images using nsOCT takes approximately 120 seconds.

## 4. Conclusion

In this paper, we demonstrated how adaptations of nsOCT such as sf-cmOCT, and a new method looking at ASFP differences, can show an increase in sensitivity to detect nanoscale changes occurring within the cornea than previously reported. For sf-cmOCT, by taking a reference ASFP and correlating it with all other profiles found at the same time point, the internal order of the sub-micron structure was visualised through mapping the Pearson correlation coefficient. When looking at the new nsOCT method based on calculating the difference between ASFPs, more qualitative and quantitative information was extracted. By taking the difference between a healthy reference profile and a damaged profile, the new profile formed gave comprehensive information about the nanoscale changes occurring within the cornea. We can now monitor features such as the structural disorder of the stroma using correlation methods and collect information about the variations in spatial period profiles relating to the nanoscale structures in tissue with better sensitivity. Further work is required to develop and adapt this method for specific applications, as well as to establish appropriate visualisation approaches for the generated profiles. The changes in macroscopic structure were also investigated using conventional OCT, and the increase in corneal thickness was calculated through dynamic processing edge detection. In the context of corneal injury and repair, this method investigates the optical spatial periods present within the cornea. It would require accurate measurements of the refractive index changes between each layer of the cornea to get information about physical structures when necessary. There are also limitations present for nsOCT in clinical settings, and efforts are needed to create stable experimental set-ups for accurate measurements, particularly through the depth of the cornea. The results presented in this paper help to demonstrate the potential of nsOCT to be used as a clinical *in vivo* tool to monitor nanoscale changes within the sub-micron structure of the cornea, as well as in other biological tissues.

## Supplemental information

Supplement 1 Supplemental material containing additional PBS analysis https://doi.org/10.6084/m9.figshare.32613570

## Data Availability

Data underlying the results presented in this paper are not publicly available at this time but may be obtained from the authors upon reasonable request.

## Supplemental document

See Supplement 1 for supporting content.
