# Supplementary material for: Nanosensitive optical coherence tomography to monitor corneal burn and treatment response in vivo
Source: Biomed Opt Express. 2026 Jun 12;17(7):3592–608. doi: 10.1364/BOE.599649 (PMC13372376; doi:10.1364/BOE.599649)
Supplement: Supplementary file 1 [file boe-17-7-3592-s001.pdf]

Supplemental Document

Biomedical Optics EXPRESS

Nanosensitive optical coherence tomography to monitor corneal burn and treatment response in vivo: supplement

EANNA JOHNSTON,[1] SERGEY ALEXANDROV,[1] RAJIB DEY,[1] ELLEN DONOHOE,[2,3] AOIFE CANNING,[2,3] THOMAS RITTER,[2,3] AND MARTIN LEAHY[1,3,*]

[1] *Tissue Optics and Microcirculation Imaging (TOMI) Facility, School of Natural Sciences, University of Galway, Galway, Ireland*
[2] *Regenerative Medicine Institute (REMEDI), Discipline of Advanced Therapeutics, School of Medicine, University of Galway, Galway, Ireland*
[3] *CURAM, SFI Research Centre for Medical Devices, University of Galway, Galway, Ireland*
[*] *martin.leahy@universityofgalway.ie*

This supplement published with Optica Publishing Group on 12 June 2026 by The Authors under the terms of the Creative Commons Attribution 4.0 License in the format provided by the authors and unedited. Further distribution of this work must maintain attribution to the author(s) and the published article's title, journal citation, and DOI.

Supplement DOI: https://doi.org/10.6084/m9.figshare.32613570

Parent Article DOI: https://doi.org/10.1364/BOE.599649

NANOSENSITIVE OPTICAL COHERENCE TOMOGRAPHY TO MONITOR CORNEAL BURN AND TREATMENT RESPONSE IN VIVO:SUPPLEMENTAL DOCUMENT

DYNAMIC PROCESSING EDGE DETECTION

To obtain accurate thickness measurements of the cornea, an edge detection method based on OCT layer segmentation was used [1]. The image is first converted into a cost map where pixels with strong axial intensity gradients have low cost and homogeneous regions have high cost. Each pixel is treated as a node in a graph, with edges allowed only between neighboring A-lines and within a limited vertical range, enforcing surface smoothness. A dynamic-programming shortest-path search then finds the minimum-cost left-to-right path, which corresponds to a continuous anatomical boundary. The mean distance between the anterior and posterior surface is then taken as the corneal thickness. This is then repeated for all B-frames in the OCT volume to give a mean thickness measurement for the cornea. A flow chart of this process can be seen in S1.

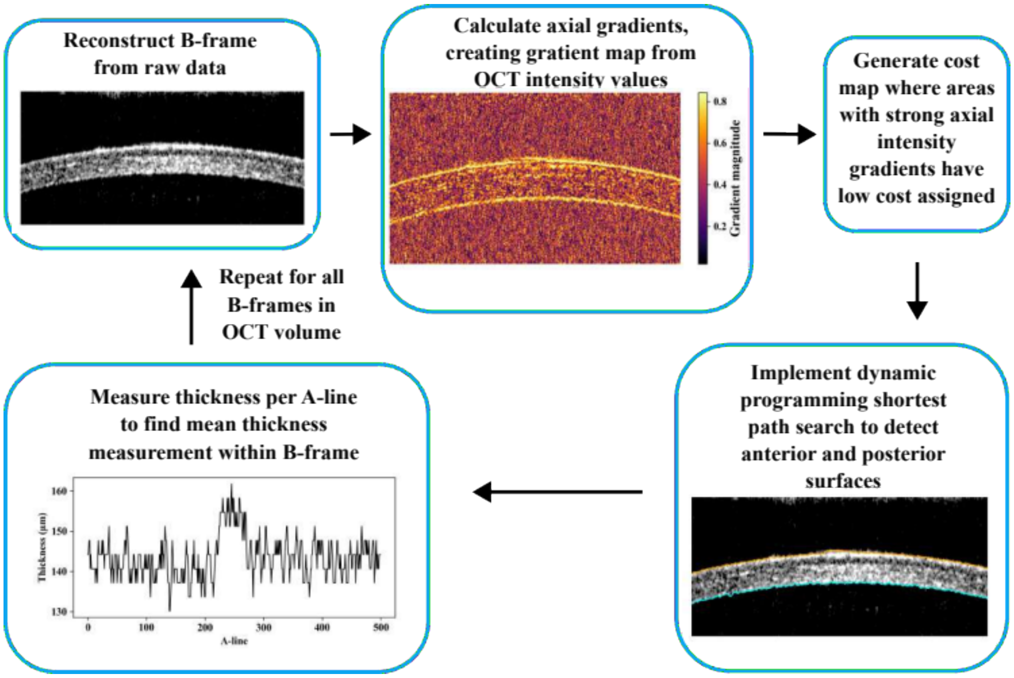

Fig. S1. Flow chart for edge detection algorithm and thickness measurement for OCT volumes

PBS CONTROL CORNEAL BURN SAMPLES

Here, we show results generated from PBS-treated control samples to help validate the nsOCT processing methods presented. When looking at the sf-cmOCT results for PBS-treated samples, the correlation coefficients remain low compared to the healthy cornea, indicating that by day 7, the structure remains disordered. The plots in figure S2 show this downward trend in correlation coefficients, with statically significant differences ($p < 10-10$) between the healthy, day 1, and day 3 corneas. The corneas by day 7 show no statistically significant differences

(p > 0.05) with day 3. Table S2 shows the correlation coefficient values for all samples treated with PBS, and how the correlation coefficient for all samples follows the same trend.

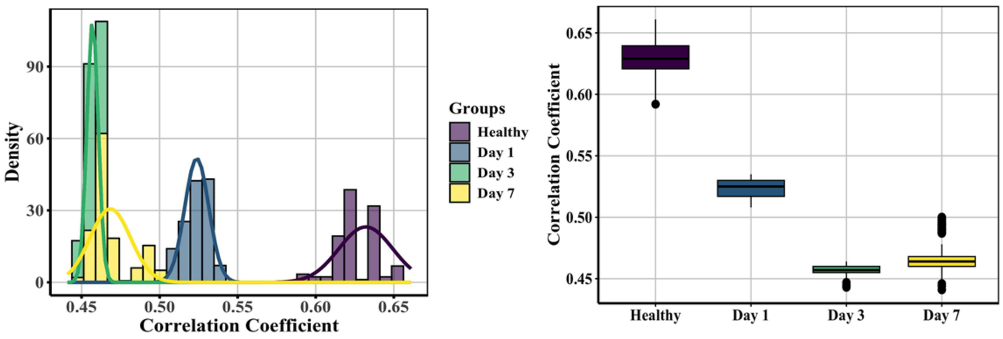

Fig. S2. Pearson correlation coefficient distribution for PBS control group.

The presented nsOCT method based on calculating the difference between axial spatial frequency profiles (ASFPs) was also validated using PBS samples. It was found that by day 7, the difference in axial spatial frequency contributions between the healthy profile was still high and comparable to the difference found at day 1 of treatment. Figure S3 shows how during the PBS control treatment, the median ASFP power distribution remains high as treatment reaches day 7. When looking through the depth of the cornea in figure S4, the day 1 and 7 samples have no clear separation in mean spatial frequency power, and the standard deviations remain high. Table S4 shows the ASFP power values for all samples treated with PBS, and how they increase over time.

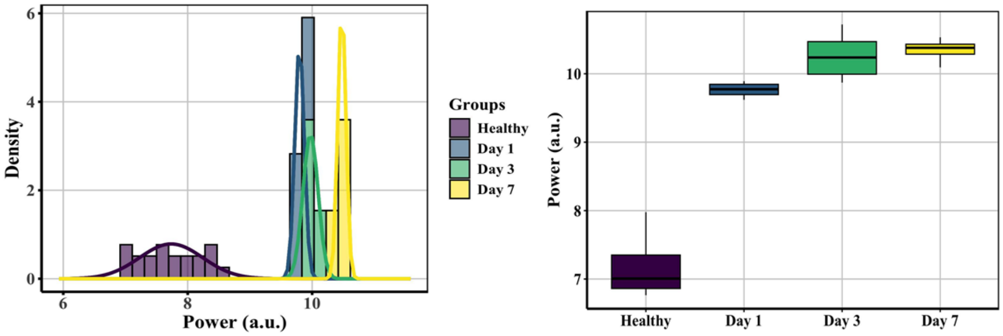

Fig. S3. Pearson correlation coefficient distribution for PBS control group.

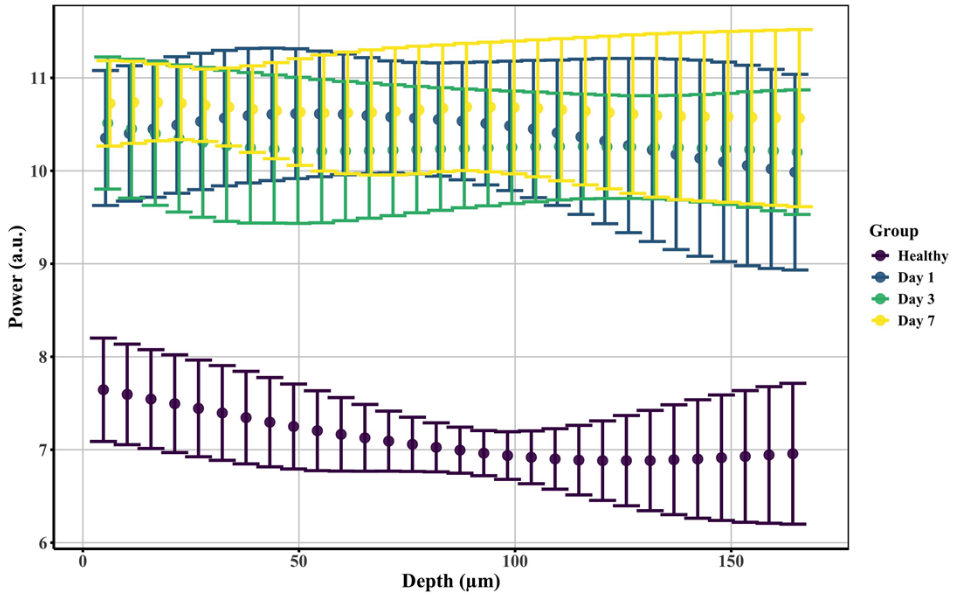

Fig. S4. Mean ASFP power value and standard deviation of en-face images through cornealdepth of PBS treated samples.

TABLE OF VALUES FOR ALL SAMPLES

This section contains all the mean correlation coefficient and ASFP power values with standard deviations for both nsOCT methods presented for all MSC-EV and PBS-treated corneas.

Table S1. Mean correlation coefficient and standard deviation values for MSC-EV-treated samples

Sample	Day 1	Day 3	Day 7	D7 – D1	D7 – D3
**S1**	0.488 ± 0.007	0.573 ± 0.012	0.646 ± 0.012	+0.158	+0.073
**S2**	0.486 ± 0.003	0.577 ± 0.010	0.642 ± 0.003	+0.156	+0.065
**S3**	0.486 ± 0.007	0.579 ± 0.009	0.647 ± 0.007	+0.161	+0.068
**S4**	0.487 ± 0.004	0.587 ± 0.006	0.648 ± 0.005	+0.161	+0.061
**S5**	0.479 ± 0.002	0.582 ± 0.012	0.647 ± 0.004	+0.168	+0.065
**S6**	0.478 ± 0.003	0.584 ± 0.010	0.651 ± 0.003	+0.173	+0.067
**S7**	0.480 ± 0.006	0.589 ± 0.009	0.648 ± 0.003	+0.168	+0.059

Table S2. Mean correlation coefficient and standard deviation values for PBS-treated samples

Sample	Day 1	Day 3	Day 7	D7 – D1	D7 – D3
**S1**	0.530 ± 0.005	0.458 ± 0.005	0.465 ± 0.003	−0.065	+0.007
**S2**	0.520 ± 0.009	0.458 ± 0.002	0.460 ± 0.006	−0.060	+0.002
**S3**	0.524 ± 0.006	0.460 ± 0.003	0.494 ± 0.004	−0.030	+0.034
**S4**	0.524 ± 0.009	0.460 ± 0.002	0.461 ± 0.006	−0.063	+0.001
**S5**	0.521 ± 0.009	0.451 ± 0.005	0.460 ± 0.006	−0.061	+0.009

Table S3. Mean difference in ASFP power and standard deviation values for MSC-EV-treated samples

Sample	Day 1	Day 3	Day 7	D7 – D1	D7 – D3
**S1**	8.98 ± 0.75	9.20 ± 0.86	9.98 ± 0.78	+1.00	+0.78
**S2**	9.50 ± 0.82	9.47 ± 1.60	9.12 ± 1.24	−0.38	−0.35
**S3**	10.22 ± 1.44	9.15 ± 1.23	8.99 ± 1.20	−1.23	−0.16
**S4**	9.94 ± 1.39	9.23 ± 1.23	9.20 ± 1.38	−0.74	−0.03
**S5**	9.19 ± 1.07	10.34 ± 1.45	10.14 ± 1.45	+0.95	−0.20
**S6**	9.58 ± 1.24	10.34 ± 1.49	9.90 ± 1.38	+0.32	−0.44
**S7**	10.03 ± 1.33	10.35 ± 1.36	9.76 ± 1.58	−0.27	−0.59

Table S4. Mean difference in ASFP power and standard deviation values for PBS-treated samples

Sample	Day 1	Day 3	Day 7	D7 – D1	D7 – D3
**S1**	9.42 ± 1.13	9.97 ± 1.24	10.55 ± 1.59	+1.13	+0.58
**S2**	9.63 ± 1.71	10.40 ± 1.45	10.20 ± 1.29	+0.57	−0.20
**S3**	9.75 ± 1.35	10.32 ± 1.54	11.20 ± 1.74	+1.45	+0.88
**S4**	10.07 ± 1.53	10.58 ± 1.32	10.11 ± 1.73	+0.04	−0.47
**S5**	9.27 ± 1.23	8.87 ± 1.10	10.37 ± 1.82	+1.10	+1.50

References

1. M. K. Garvin, M. D. Abramoff, X. Wu, et al., "Automated 3-D Intraretinal Layer Segmentation of Macular Spectral-Domain Optical Coherence Tomography Images," IEEE Transactions on Med. Imaging 28, 1436–1447 (2009).
